# Copper Quantum Dot/Polyacrylamide Composite Nanospheres: Spreading on Quartz Flake Surfaces and Displacing Crude Oil in Microchannel Chips

**DOI:** 10.3390/polym16081085

**Published:** 2024-04-12

**Authors:** Xinru Ma, Haien Yang, Xiaofei Liu, Lixiang Zeng, Xinzi Li, Lijun Zheng, Yu Yang, Lei Cao, Weikai Meng, Junping Zheng

**Affiliations:** 1 Tianjin Key Laboratory of Composite and Functional Materials, School of Material Science and Engineering, Tianjin University, Tianjin 300072, China; maxinru16tju@163.com (X.M.); li_xz@tju.edu.cn (X.L.); yangytju@163.com (Y.Y.); charlie20231226@163.com (L.C.); mengwk2022@163.com (W.M.); jpzheng@tju.edu.cn (J.Z.); 2 Xi’an Changqing Chemical Industry Group Co., Ltd., Xi’an 710021, China; yhe_cq@petrochina.com.cn (H.Y.); zlixiang_cq@petrochina.com.cn (L.Z.); zhlj_cq@petrochina.com.cn (L.Z.)

**Keywords:** composite nanospheres, polyacrylamide, copper quantum dot, spreading behavior, simulated displacement

## Abstract

Polyacrylamide, silica, and other nanoparticles have all been realized in the field of enhanced oil recovery. Researchers often explore the mechanisms of spreading behavior and simulated displacement to develop more efficient types of nanoparticles. In this study, copper quantum dots were introduced into a acrylamide copolymerization system to obtain composite nanospheres and its structure, topographic, and application performance were characterized. The results show that the composite nanospheres have a particle size of around 25 nm, are uniformly loaded with copper particles, and have good temperature resistance. The spreading ability on the quartz flake surfaces and displacement effect in microchannels of composite nanospheres, acrylamide copolymer nanospheres, and copper quantum dots were compared by nanofluid spreading experiments and microchannel chip oil displacement experiments. The results indicate that the composite nanospheres can effectively reduce the water contact angle, promote the spreading of aqueous phase, and accelerate the oil droplet removal process; the accelerating effect is stronger than other samples. Its oil displacement effect is also the strongest, and it is minimized by the influence of channel size, temperature, and dispersing medium, with better stratigraphic adaptability. This work supports the practical application of copper quantum dot/polyacrylamide composite nanospheres in the oilfield.

## 1. Introduction

Nanoparticles refer to particles smaller than 100 nm in some one dimension, which yield important scientific research value due to their special physical properties [1,2]; whereas quantum dots are even smaller in size, generally below 10 nm [3], and nanofluids are defined as dispersed systems formed by a suspension of nanoparticles in a base fluid [4]. Nanofluids give rise to potential applications in various fields of industry, including solar collectors [5,6], lubrication [7,8], and oil recovery [9,10,11], due to the enhancement of various properties, such as thermal conductivity [12,13], electrical conductivity [14,15], and viscosity [16,17]. In fact, such nanoparticle suspension systems are also common in nature, such as raindrops on oil-stained pavements, and we can observe the interaction of such complex liquid systems with solid surfaces. The interaction of liquid droplets with surfaces has attracted a great deal of interest from researchers.

In previous years, it has been shown that the spreading and wetting behavior of nanofluids is altered when compared to a base fluid without nanoparticles. The study by Wasan and Nikolov [18,19,20] found that suspended particles will exhibit ordering within the three-phase wedge region formed by edge confinement during nanofluid diffusion, and the structural disjoining pressure generated by orderly arrangement will enhance the diffusion behavior of the nanofluid. Vafaei et al. [21] observed that the contact angle of the nanofluid with substrates of different properties is sensitive to both nanoparticle concentration and nanoparticle size. The above studies suggest that nanofluids are expected to be applied in enhanced oil recovery (EOR) based on the properties that promote structural separation, pressure generation, and wettability modification.

The common types of nanoparticles used in EOR technology can be categorized as metal and its oxide nanoparticles [22,23,24], inorganic nanoparticles [25,26,27,28,29], and organic nanoparticles [30,31,32,33,34,35,36,37]. In his research, Shah [22] found that metal nanoparticles can reduce the viscosity of crude oil and can be used in thick oil extraction. Wang et al. [35] prepared polyacrylamide microgel nanosphere emulsion with a solid content of 35.04 wt% by two synthetic processes, which significantly reduced the oil–water interfacial tension through a synergistic mechanism with the emulsifier to achieve enhanced oil recovery. Hua et al. [36] synthesized nanoscale cross-linked polyacrylamide microspheres, investigated its plugging and oil displacement properties, and explored oil displacement mechanism in combination with visualization experiments. Wang et al. [37] prepared polymer nanospheres with nanoscale spherical and inorganic core/polymer shell structures, profile control, and oil recovery improvement experiment results show that the polymer composite microspheres can reduce the water content and significantly improve the recovery rate.

In a previous study, we synthesized acrylamide copolymer nanospheres by using acrylamide (AM), 2-acrylamide-2-methylpropanesulfonic acid (AMPS) as a monomer and N,N′-methylenebisacrylamide (MBA) as a cross-linking agent, and we chose the ammonium persulphate-sodium bisulphite ((NH_4_)_2_S_2_O_8_-NaHSO_3_) redox initiation system [38]. The increasing degree of oilfield development has put forward higher requirements on the performance of oil repellents. In this work, copper quantum dots (Cu QDs) were introduced into the above polymerization system to obtain in situ copper quantum dot/polyacrylamide composite nanospheres (Cu/PAM NPs). The molecular structure, surface morphology, and element analysis of the new composite nanospheres were characterized, and its thermal stability was tested. Combined with the nanofluid spreading experiments and microchannel chip oil displacement experiments, the spreading ability and oil displacement capacity of these new composite nanospheres were evaluated and compared with polymer nanospheres and copper quantum dots.

## 2. Experimental Section

### 2.1. Chemicals and Materials

Acrylamide (AM), 2-acrylamido-2-methylpropane sulfonic acid (AMPS), methylene-bisa-crylamide (MBA), and sodium hydroxide (NaOH) were purchased from Beijing InnoChem Science and Technology, Beijing, China. White mineral oil and sorbitan oleate (Span-80) were purchased from Tianjin Heowns Optech, Tianjin, China. Anhydrous ethanol, alkylphenol polyoxyethylene ether (TX-10), and sodium dodecyl sulfate (SDS) were purchased from Tianjin Jiangtian Chemical Industry, Tianjin, China. Acrylamide copolymer nanospheres (PAM NPs) [38] and copper quantum dot dispersion (Cu QDs) [39] were homemade. Crude oil and injected water (mineralization greater than 5000 mg·L^−1^, cations in water are mainly K^+^, Na^+^, Ca^2+^, and anions are mainly Cl^−^, F^−^, SO_4_^2−^, HCO_3_^−^ and CO_3_^2−^) were provided by Xi’an Changqing Chemical Group, Xi’an, Shanxi Province, China. Quartz flakes with specifications of 30 mm × 30 mm × 1 mm were provided by Tianjin Huaying Dingsheng Technology, Tianjin, China. The 1.0 μL microsampler was supplied by Shanghai Gaoge Industry Trade, Shanghai, China. Non-homogeneous multistage bifurcated microchannels were provided by Zhenjiang Huarui Chip Technology, Zhenjiang, Jiangsu Province, China.

### 2.2. Synthesis of Copper Quantum Dot/Polyacrylamide Composite Nanospheres (Cu/PAM NPs)

White mineral oil, surfactant Span-80, and TX-10 were added sequentially in a four-necked flask and mixed well under mechanical stirring. Then, appropriate amounts of AM, AMPS, and MBA were sequentially weighed and added to the beaker, and an appropriate amount of copper quantum dots dispersion was added and stirred until complete dissolution. The pH was adjusted by adding a quantity of NaOH solution and the aqueous phase was formed. Subsequently, the aqueous phase was slowly added to the oil phase, and the emulsion was obtained after complete addition and continued stirring for a period of time to fully emulsify. Then, nitrogen was passed and the temperature was raised to initiate polymerization. After the end of the self-heating phenomenon, the temperature was kept the same for a certain amount of time to obtain a light yellow uniform and stable emulsion, that is, copper quantum dot/polyacrylamide composite nanosphere (Cu/PAM NPs) emulsion.

**Figure 1:**
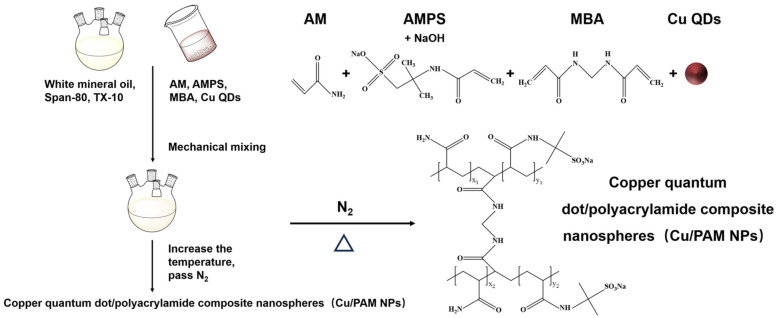
Synthesis of copper quantum dot/polyacrylamide composite nanospheres.

A certain amount of ethanol and a small amount of emulsion was slowly added (mass ratio of ethanol to emulsion is 10:1) to the beaker, followed by demulsification and precipitation, even dispersal in ultrasonic oscillators (KQ5200, Kunshan Supmile, Kunshan, China), centrifuging in a table high-speed centrifuge (H1850, Xiangyi Instrument, Changsha, China). The precipitation was placed into a vacuum oven (DZF6020, Bangxi Instrument, Shanghai, China) at 60 °C for 12 h to obtain a white powdered solid, that is, copper quantum dot/polyacrylamide composite nanospheres (Cu/PAM NPs) dry powder. The synthesis scheme is shown in Figure 1.

### 2.3. Characterization

The samples were prepared by the potassium bromide compression method, and the infrared absorption spectra of copper quantum dot/polyacrylamide composite nanospheres were tested using an Avatar-360 Fourier infrared spectrometer (Thermo Fisher Scientific, Waltham, MA, USA), and their molecular structures were analyzed.

The emulsion was diluted in n-Hexane at a certain ratio, transferred to a quartz cuvette, and the particle size and its distribution of the copper quantum dot/polyacrylamide composite nanospheres was examined using a Zetasizer Nano ZS laser particle sizer (Malvern Instruments, Malvern, UK).

The emulsion was diluted in n-hexane at a certain ratio, or dry powder dispersed at a certain proportion in ethanol, mixed well, and then a sample drop was taken on the molybdenum mesh with a pipette gun. The surface morphology of copper quantum dots/polyacrylamide composite nanospheres was observed using JEM-2100F transmission electron microscope (Japan Electron Optics Laboratory, Tokyo, Japan) with an operating voltage of 120 kV and a point resolution of 0.23 nm, and its elemental distribution images were obtained by surface scanning using Oxford Aztec X-Max 80T energy dispersive X-ray spectrometer (Oxford Instruments, Oxford, UK) with an operating voltage of 200 kV and a resolution better than 127 eV.

The appropriate amount of dry powder was taken in a polytetrafluoroethylene beaker, nitric acid was added and dissolved at high temperature on an electric hot plate, the sample was completely dissolved and then fixed, and the dilution times were calculated. The copper concentration of the solution was determined by an Agilent 5110 (Agilent Technologies, Santa Clara, CA, USA) inductively coupled plasma optical emission spectrometer (ICP-OES), and the copper content in the copper quantum dot/polyacrylamide composite nanospheres was calculated.

Thermogravimetric analysis was carried out on an instrument of the type TG 209 F3Tarsus (NETZSCH, Selb, Germany) in a nitrogen atmosphere with a temperature rise speed of 10 °C/min and a temperature upper limit of 800 °C.

### 2.4. Nanofluid Spreading Experiment

**Figure 2:**
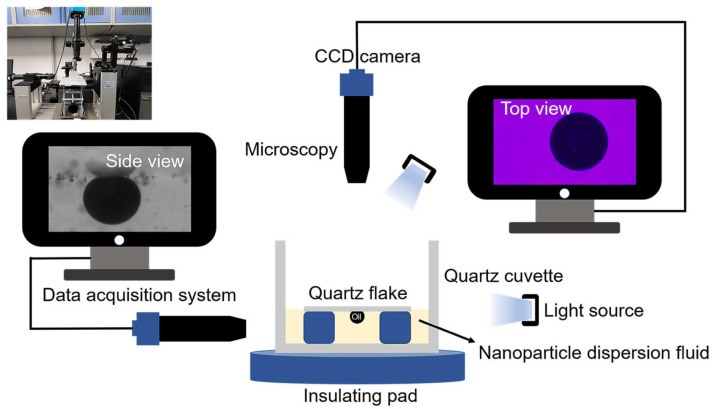
Apparatuses of nanofluid spreading experiment.

Three samples of 0.1 wt% copper quantum dot/polyacrylamide composite nanosphere dry powder dispersion, 0.1 wt% acrylamide copolymer nanospheres dry powder dispersion, and 100 ppm copper quantum dot dispersion were configured, respectively, SDS was added to the deionized water and the three solutions mentioned above to form a compounded system containing 6 mmol/L SDS. A 1.0 μL of crude oil was transferred onto the surface of a quartz flake using a micro feeder, and the loaded crude oil side was placed face down into a quartz cuvette, and the sample was slowly added to the vessel until it touched the crude oil and the quartz flake, and the process of nanofluid spreading and crude oil removal was observed by the device constructed, referring to the work presented in [20]. The device diagram is shown in Figure 2 (Surface tension meter (OCA15EC, German Dataphysics Company, Stuttgart, Germany), CCD camera (UCMOS14000KPA, Japan Sony Corporation, Tokyo, Japan), industrial digital microscope (BC, Bosheng Electronic Technology, Dongguan, China).

### 2.5. Microchannel Chip Oil Displacement Experiment

**Figure 3:**
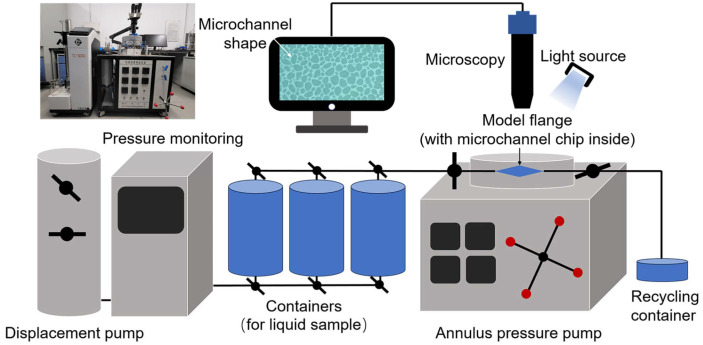
Apparatuses of microchannel chip oil displacement experiment.

A non-homogeneous multistage bifurcation microchannel model was established, and two scales of 50–200 μm and 5–50 μm inner diameter of the pore channel were chosen to simulate the large and small pore throats of the formation. A 5‰ copper quantum dot/polyacrylamide composite nanospheres emulsion dispersion, a 5‰ acrylamide copolymer nanosphere emulsion dispersion, and a 100 ppm copper quantum dot dispersion were configured and left to stand for fourteen days at an ambient temperature, and then microchannel chip oil displacement experiments were performed to compare the displacement efficiencies of the three samples at different channel scales, temperatures, and dispersing media conditions. The experiments were set up with an annulus pressure of 0.5 MPa and an injection pressure of 0.25 MPa. Crude oil was injected into the microchannel chip using the constant pressure mode of the displacement pump (TC-100D, Jiangsu Tuochuang Scientific Research Instrument, Haian, China), and when the crude oil was full, the sample was injected in the same manner, and the dynamic evolution of the oil and water was observed using a microscope (Z6 APO, Germany Leica Instrument, Vizsla, Germany), and real-time images were taken. Processing the images before and after the experiment, the real-time image was segmented into channel histograms of red, blue, and green colors. The blue channel histogram, with the most obvious color difference between the crude oil and other parts was selected, and then converted into a grayscale image using Image J software (Fiji). The crude oil area and displacement efficiency are calculated to carry out a comprehensive analysis of the oil displacement ability of different samples. Figure 3 shows the schematic diagram of the microchannel chip oil displacement experiment device.

## 3. Results and Discussion

### 3.1. Structural Characterization

**Figure 4:**
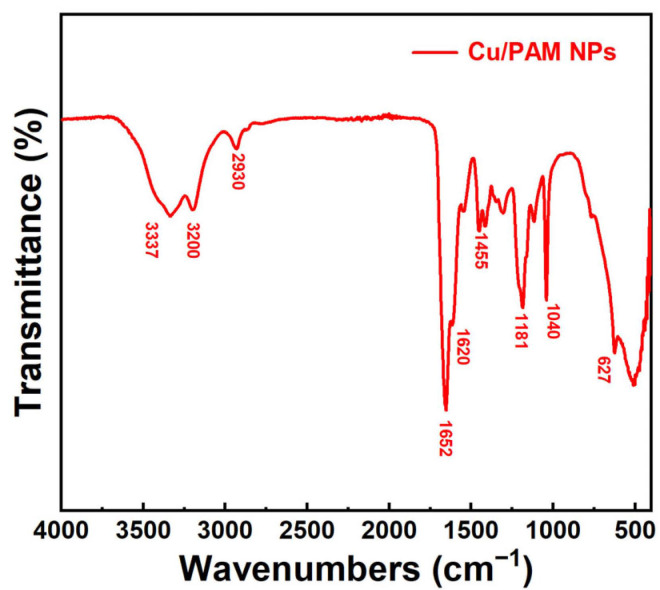
FT-IR spectra of the copper quantum dot/polyacrylamide composite nanospheres.

Figure 4 illustrates the FT-IR spectra of copper quantum dot/polyacrylamide composite nanospheres. In the figure, the medium intensity peaks at 3200 cm^−1^ and 3337 cm^−1^ correspond to the symmetrical and asymmetrical stretching vibration of N-H, respectively, which are the characteristic peaks of primary amines. The peaks at 1652, 1620, 1455, 1181, and 627 cm^−1^ pertain to C=O stretching, N-H bending, C-N stretching, N-H in-plane rocking, and N-H out-of-plane rocking vibration, respectively, which confirmed the presence of amide groups. The peak at 1040 cm^−1^ can be assigned to the S-O stretching vibration, which was the characteristic peak of the sulphonic acid group. The absence of characteristic peaks of C=C in the FT-IR spectra confirms that the polymerization reaction of the monomers AM and AMPS was successful.

### 3.2. Topographic Representation

**Figure 5:**
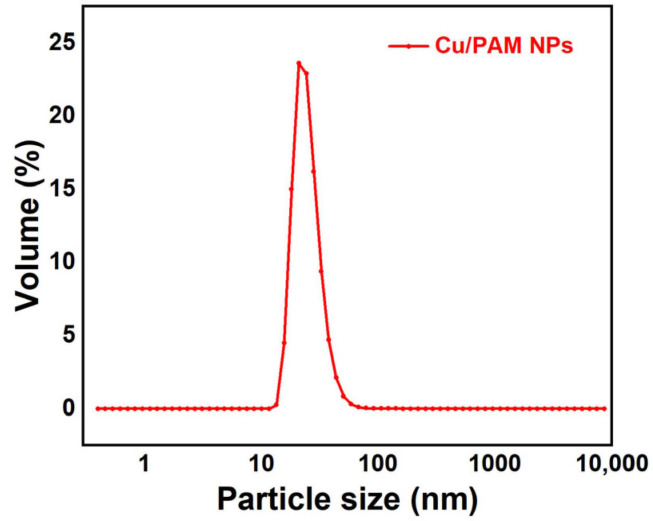
Particle size chart of copper quantum dot/polyacrylamide composite nanospheres.

**Figure 6:**
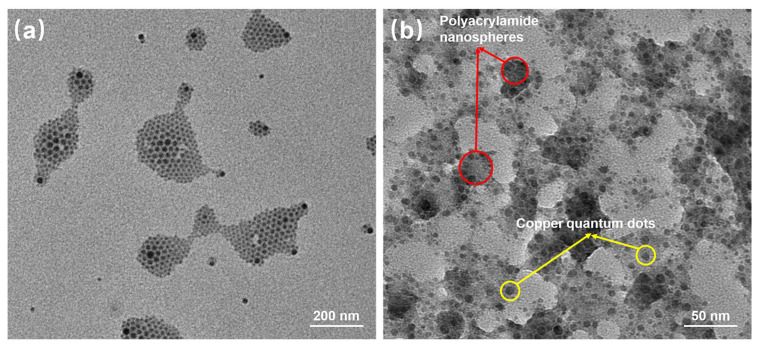
TEM of copper quantum dot/polyacrylamide composite nanospheres: (**a**) Emulsion; (**b**) Dry powder.

**Figure 7:**
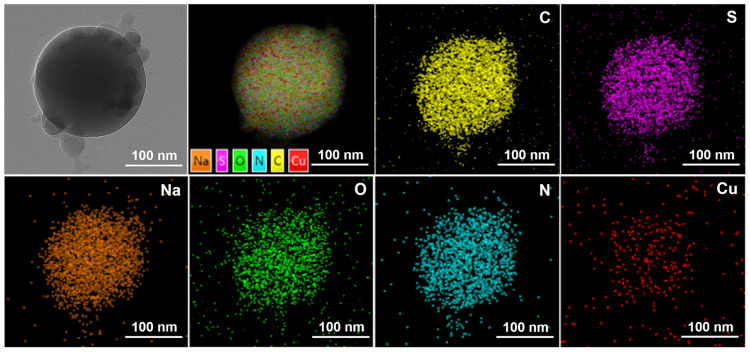
TEM-EDS of copper quantum dot/polyacrylamide composite nanospheres powder.

Figure 5 reflects an average particle size of 25 nm for the copper quantum dot/polyacrylamide composite nanospheres. Figure 6a,b shows the morphology of the copper quantum dot/polyacrylamide composite nanospheres emulsion and powder under TEM, respectively. Figure 6a shows the regular spherical morphology of copper quantum dot/polyacrylamide with a smooth particle shape and more uniform particle distribution, which matches the results shown by the laser particle size meter. The microsphere particles in Figure 6b, although appearing partially adherent, show more clearly the uniform distribution of copper nanoparticles in the composite nanospheres. The composite nanospheres were also analyzed using energy dispersive X-ray spectroscopy (EDS) to confirm their elemental composition. The TEM-EDS energy spectra of the composite nanospheres are shown in Figure 7, which strongly depicts the presence of the elements C, O, N, S, Na and Cu in the prepared nanocomposites, confirming the successful copolymerization of the AM, AMPS, and copper quantum dots. The elemental Cu content in the composite nanospheres was tested using atomic emission spectroscopy and the results were obtained as shown in Table 1, where the copper content in the emulsion and dry powder was about 200 ppm and 39 ppm, respectively, which is in accordance with the solid content of the emulsion of the composite nanospheres, and proves that the copper nanoparticles are fully incorporated into the nanosphere structure.

### 3.3. Stability Analysis

**Figure 8:**
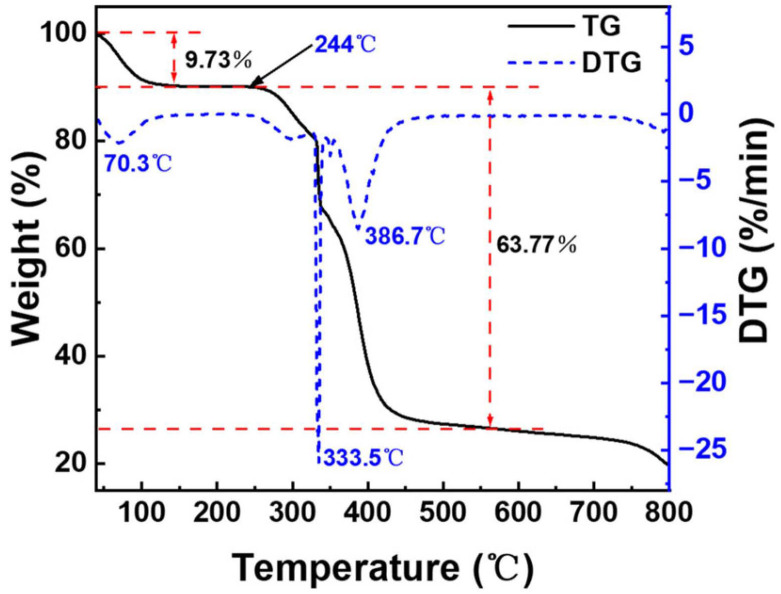
TG and DTG of the copper quantum dot/polyacrylamide composite nanospheres.

Figure 8 represents the TG and DTG curves of the copper quantum dot/polyacrylamide composite nanospheres, in which four phases with different weight loss rates can be observed in this thermogram. The first stage is 40–244 °C and the sample loses 9.73% of its weight at this stage, which is mainly due to the volatilization of water molecules adsorbed by the hydrophilic amide groups in the sample. The second stage is 244–328 °C and the weight loss of the samples is 8.56% in this stage, which is attributed to the decomposition of the amide groups. The third stage is 328–350 °C and the weight loss rate of the samples is higher in this stage. The DTG curve peaked at 333.5 °C, and the weight loss reached 16.62%, which is mainly caused by the decomposition of the sulfonic acid group of the composite nanospheres and the partial breakage of the molecular chain. The fourth stage was 350–560 °C, and the thermal decomposition of the main chain of the composite nanospheres resulted in a weight loss of 38.59%. From the analysis of the thermal weight loss curve, it can be seen that the decomposition of the composite nanospheres mainly occurs above 244 °C, which means that the sample has good thermal stability.

### 3.4. Nanofluid Spreading Experiment

**Figure 9:**
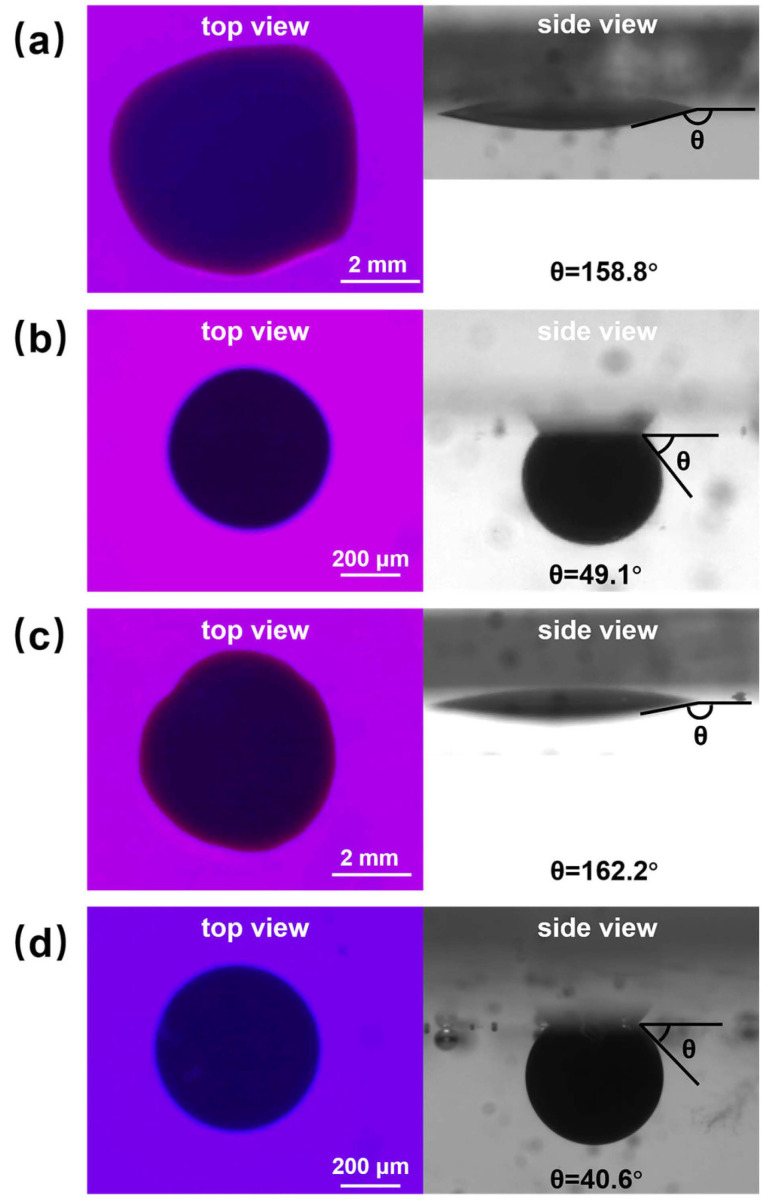
Images of 0.1 wt% copper quantum dot/polyacrylamide composite nanosphere dry powder dispersion at different temperatures for nanofluid spreading experiments: (**a**) 25 °C, experimental initial plots; (**b**) 25 °C, experimental 60 min effect plots; (**c**) 65 °C, experimental initial plots; (**d**) 65 °C, experimental 60 min effect plots.

Figure 9 shows the initial and 60 min effect plots of nanofluid spreading experiments with 0.1 wt% copper quantum dot/polyacrylamide composite nanosphere dry powder dispersion at different temperatures.

From the top view in Figure 9, it can be seen that the spreading process of composite nanospheres can round the crude oil droplets at 25 °C and 65 °C. As can be seen from the side view, the spreading process of composite nanospheres can result in a significant reduction in the water contact angle (the angle between the aqueous phase and quartz flake), which is calculated using image J software, which revealed that the contact angle is increased from 158.8° to 49.1° at 25 °C, and from 162.2° to 40.6° at 65 °C. It indicates that, although the spreading behavior of copper quantum dot/polyacrylamide composite nanosphere dry powder dispersion on the solid phase cannot completely remove the crude oil droplets from the quartz flake, it can break the adhesion between the crude oil droplets and the quartz flake through the Brownian motion, promote the wetting of the aqueous phase, and increase the contact angle of the oil phase and the solid phase. The elevated temperature is conducive to the enhancement of this effect.

The effect of the acrylamide copolymer nanospheres on the water contact angle during the spreading process is not as significant as that of composite nanospheres, and the contact angle decreased to 89.5° and 61.8° under the conditions of 25 °C and 65 °C, respectively. The spreading behavior can make the crude oil droplets round only at 65 °C, and the experimental images are shown in Figure S1. The spreading process of the copper quantum dots can only shrink the crude oil to some extent, and has little effect on the removal of crude oil, and the experimental real-time images are shown in Figure S2.

**Figure 10:**
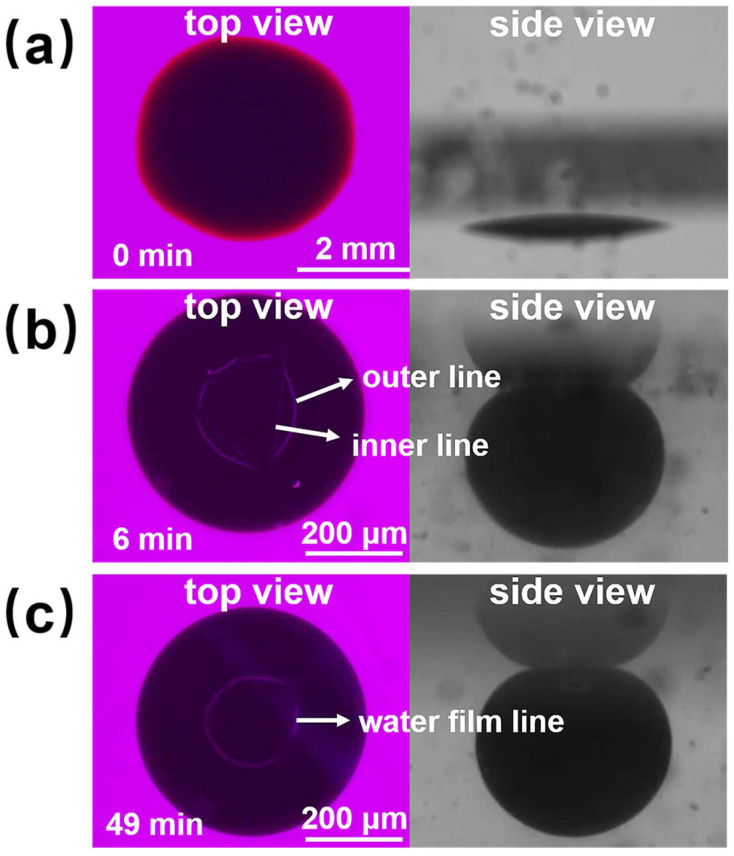
Schematic of the experimental procedure for nanofluid spreading experiments with 6 mmol/L SDS solution at 25 °C. (**a**) Experimental initial plots; (**b**) Experimental 6 min effect plots; (**c**) Experimental 49 min effect plots.

Figure 10 shows the process diagram of nanofluid spreading experiments with 6 mmol/L SDS solution at 25 °C. At the sixth minute of the experiment, the outer and inner contact lines appeared, and the area between the outer and inner contact lines was the water film formed by the sample between the crude oil and the quartz sheet, and the inner edge of the water film moved inward at a faster rate until the inner contact line disappeared at 49 min. A complete water film formed and a water film line appeared, proving that the oil droplets had been removed. The mechanism of oil droplet stripping during this process is consistent with a combination of the “rolling-up” mechanism and the “diffusional” mechanism [40].

**Figure 11:**
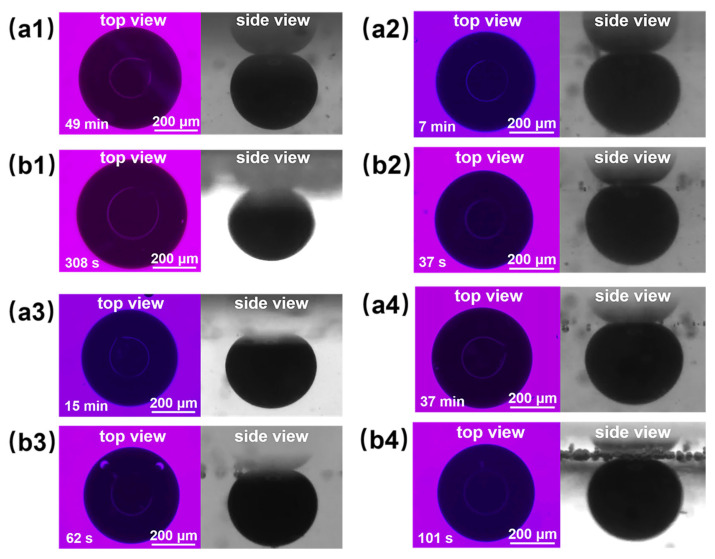
Final effect plots of nanofluid spreading experiments with different systems at (**a**) 25 °C and (**b**) 65 °C: (**a1**,**b1**) 6 mmol/L SDS; (**a2**,**b2**) 0.1 wt% copper quantum dot/polyacrylamide composite nanosphere dry powder dispersion containing 6 mmol/L SDS; (**a3**,**b3**) 0.1 wt% acrylamide copolymer nanosphere dry powder dispersion containing 6 mmol/L SDS; (**a4**,**b4**) 100 ppm copper quantum dot dispersion containing 6 mmol/L SDS.

Figure 11(a2,b2) shows the final effect diagrams of nanofluid spreading experiments with 0.1 wt% composite nanosphere dry powder dispersion containing 6 mmol/L SDS at 25 °C and 65 °C. The crude oil removal times of this sample were at 7 min and 37 s, respectively, which were 85.71% and 87.99% shorter than that of the SDS solution under the same conditions, confirming that the composite nanospheres have an obvious accelerating effect on the crude oil removal.

Figure 11 and Table 2 demonstrate the final effect diagrams and the time required to remove crude oil for the nanofluid spreading experiments with different systems at 25 °C and 65 °C. The comparison reveals that the system with copper quantum dot/polyacrylamide composite nanospheres and SDS removes crude oil droplets in a significantly shorter period of time than the other systems, suggesting that the composite nanospheres had the most pronounced accelerating effect on the process of oil droplet removing.

**Figure 12:**
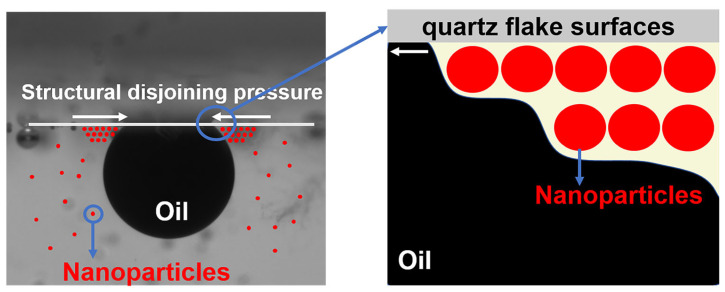
Schematic mechanism of nanoparticles for enhanced fluid wetting and crude oil removal.

The accelerating effect of nanoparticles on the oil droplet removal process may be due to the structural separation pressure gradient created by their ordered arrangement at the three-phase interface [18,41], as shown in Figure 12. The higher tension at the tip of the wedge region drives the diffusion of the nanofluid towards its tip and the inward movement of the three-phase contact line, enhancing the dynamic spreading behavior of the nanofluid.

The disjoining pressure gradient is a combination of van der Waals and electrostatic forces [42], the magnitude of which may be related to the type of nanoparticles. The introduction of a small amount of copper quantum dots effectively enhances the electrostatic force between the polymer-based nanospheres, enabling the composite nanospheres to produce a larger disjoining pressure gradient compared to the polymer nanospheres at the same concentration. The magnitude of the disjoining pressure gradient is also related to the concentration of the nanoparticles [43]. Due to the lower concentration of copper quantum dots, the disjoining pressure gradient formed is also smaller, and the copper quantum dots are more prone to displaying agglomeration behavior than the other two nanoparticles [44], thus, weakening its effect.

### 3.5. Microchannel Chip Oil Displacement Experiment

**Figure 13:**
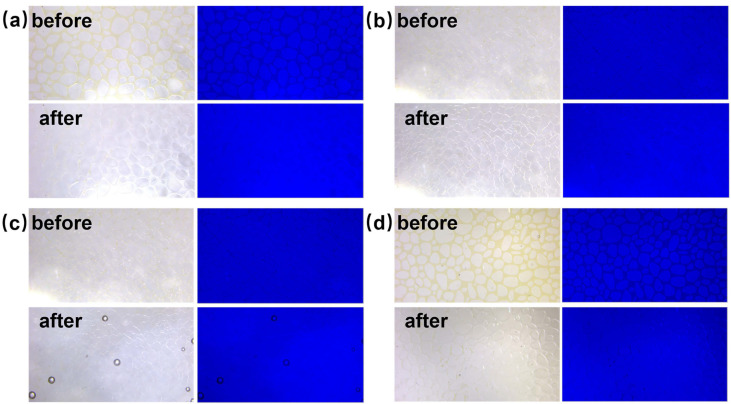
Real-time images and blue channel histograms before and after microchannel chip oil displacement experiments with 5‰ copper quantum dot/polyacrylamide composite nanosphere emulsion dispersion at different experimental conditions. (**a**) Pore inner diameter of 50–200 μm, temperature of 25 °C, dispersing medium of deionized water. (**b**) Pore inner diameter of 5–50 μm, temperature of 25 °C, dispersing medium of deionized water. (**c**) Pore inner diameter of 5–50 μm, temperature of 65 °C, dispersing medium of deionized water. (**d**) Pore inner diameter of 50–200 μm, temperature of 25 °C, dispersing medium of injected water.

Figure 13 shows the real-time images and the blue channel histograms before and after the microchannel chip oil displacement experiments with 5‰ copper quantum dot/polyacrylamide composite nanosphere emulsion dispersion at different channel scales, temperatures, and dispersing media, respectively, and the images clearly show that the samples can have a good displacement effect under different experimental conditions. Acrylamide copolymer nanosphere emulsion dispersion and copper quantum dot dispersion showed a significant decrease in the displacement effect at different experimental conditions, and its experimental effects are shown in Figures S3–S6. Table 3, Table 4, Table 5 and Table 6 show the corresponding data of the oil film area and displacement efficiency.

From the data in Table 3 and Table 4, the oil displacement efficiency at both channel scales in descending order were copper quantum dot/polyacrylamide composite nanospheres, copper quantum dots, and acrylamide copolymer nanospheres at 25 °C and the dispersion medium of deionized water, in which the oil displacement efficiency of the copper quantum dot/polyacrylamide composite nanospheres could reach 96.42% and 94.68%, respectively. It was found that the oil displacement efficiency of several samples in the small channel was reduced. Among them, the acrylamide copolymer nanospheres exhibited the largest decrease in displacement efficiency of 17.5 percentage points, which could be attributed to the strong hydrophilicity of the amide groups. The higher water absorption and swelling rate of the acrylamide copolymer nanospheres unloaded with copper particles [45] and the larger particle sizes led to the difficulty of their injection in the small channel. The displacement efficiency of the copper quantum dot/polyacrylamide composite nanospheres and the copper quantum dots produced the small decreases, but they still achieved 94.68% and 91.93% in the small channel, only decreasing by 1.74 and 3.71 percentage points, respectively.

Comparing the data in Table 4 and Table 5, it can be seen that the oil displacement efficiency of acrylamide copolymer nanospheres and copper quantum dot/polyacrylamide composite nanospheres were increased at 65 °C, indicating that the elevated temperature improved the oil displacement effect of nanospheres in the channel. The oil displacement efficiency of copper quantum dots was significantly reduced, probably because the elevated temperature promoted particle collision and agglomeration [46] which, in turn, affected the oil displacement effect.

By observing the data in Table 3 and Table 6, the oil displacement efficiency of several types of samples when injected water is used as the dispersing medium decreases, compared with that when deionized water is used as the dispersing medium, mainly due to the high salinity of formation water, which has high requirements for the salt resistance of the samples [47]. The copper quantum dots diminish the most in displacement efficiency, which indicates that they have poorer salt resistance. The copper quantum dot/polyacrylamide composite nanospheres have better salt resistance, which can be better adapted to the formation environment because its oil displacement efficiency is less reduced, still able to reach 95.14% in the injected water.

## 4. Conclusions

In this study, copper quantum dot/polyacrylamide composite nanospheres were prepared by reverse-phase microemulsion polymerization. Fourier transform infrared spectroscopy showed that acrylamide and 2-acrylamido-2-methylpropanesulfonic acid were successfully copolymerized. The laser particle size analyzer and transmission electron microscope results displayed that nanospheres, with a particle size of about 25 nm, uniformly loaded with copper particles were obtained. The results of energy dispersive X-ray spectra and atomic emission spectrometry test depicted that the copper quantum dots were completely incorporated into the nanosphere structure. The thermogravimetric test results indicated that the composite nanospheres have good temperature resistance and can adapt to the stratigraphic environment. The spreading on quartz flake surfaces, the effect and time required for removal crude oil of copper quantum dot/polyacrylamide composite nanospheres, acrylamide copolymer nanospheres, copper quantum dots, and related systems were compared, and the results revealed that the composite nanospheres could effectively diminish the water contact angle, and had an obvious accelerating effect on the crude oil removal. Microchannel chip oil displacement experiments compared their oil displacement effect, and the results indicated that, under the same experimental conditions, the composite nanospheres have a higher oil displacement efficiency compared to simple polymer nanospheres and copper quantum dots, and its oil displacement efficiency decreases significantly less than both in the small channel. The oil displacement efficiency of composite nanospheres can be improved by raising the temperature, while that of the copper quantum dots decreases due to the enhancement of agglomeration. The influence of the dispersing medium on the experimental results depends on the adaptability of the particles, and the composite nanospheres have the lowest oil displacement efficiency reduction and better stratigraphic adaptability. In summary, this work provides the technical basis for the practical applications of copper quantum dot/polyacrylamide composite nanospheres in the oilfield.

## Figures and Tables

**Table 1:** ICP test result of copper quantum dot/polyacrylamide composite nanospheres.

Sample	Sample Volume (g)	Calibration Volume (mL)	Test Concentration ± 0.0006 (mg/L)	Dilution Factor	Copper Content ± 0.3 (mg/L)
Emulsion	0.1110	25	0.1732	225.23	39.0
Dry powder	0.0523	25	0.4186	478.01	200.1

**Table 2:** Crude oil removal time with different systems at different temperatures.

Sample	25 °C	65 °C
SDS	49 min	308 s
Cu/PAM NPs + SDS	7 min	37 s
PAM NPs + SDS	15 min	62 s
Cu QDs + SDS	37 min	101 s

**Table 3:** Oil displacement efficiency of different samples (large channel, 25 °C, deionized water).

Sample	Initial Area	Remaining Area	Displacement Area	Displacement Efficiency (%)
Cu/PAM NPs	406,297	14,546	391,751	96.42
PAM NPs	406,297	22,590	383,707	94.44
Cu QDs	406,297	17,714	388,583	95.64

**Table 4:** Oil displacement efficiency of different samples (small channel, 25 °C, deionized water).

Sample	Initial Area	Remaining Area	Displacement Area	Displacement Efficiency (%)
Cu/PAM NPs	300,289	15,972	284,317	94.68
PAM NPs	300,289	69,242	231,047	76.94
Cu QDs	300,289	24,221	276,068	91.93

**Table 5:** Oil displacement efficiency of different samples (small channel, 65 °C, deionized water).

Sample	Initial Area	Remaining Area	Displacement Area	Displacement Efficiency (%)
Cu/PAM NPs	300,289	11,470	288,819	96.18
PAM NPs	300,289	49,332	250,957	83.57
Cu QDs	300,289	61,538	238,751	79.51

**Table 6:** Oil displacement efficiency of different samples (large channel, 25 °C, injected water).

Sample	Initial Area	Remaining Area	Displacement Area	Displacement Efficiency (%)
Cu/PAM NPs	394,111	19,160	374,951	95.14
PAM NPs	394,111	52,314	341,797	86.73
Cu QDs	394,111	91,011	303,100	76.91

## Data Availability

Data are contained within the article and Supplementary Materials.

## Supplementary Materials

The following supporting information can be downloaded at: https://www.mdpi.com/article/10.3390/polym16081085/s1. Figure S1: Images of 0.1 wt% acrylamide copolymer nanosphere dry powder dispersion at different temperatures for nanofluid spreading experiments: (a) 25 °C, experimental initial plots; (b) 25 °C, experimental 60 min effect plots; (c) 65 °C, experimental initial plots; (d) 65 °C, experimental 60 min effect plots; Figure S2: Images of 100 ppm copper quantum dot dispersion at different temperatures for nanofluid spreading experiments: (a) 25 °C, experimental initial plots; (b) 25 °C, experimental 60 min effect plots; (c) 65 °C, experimental initial plots; (d) 65 °C, experimental 60 min effect plots; Figure S3: Real-time images and blue channel histograms of microchannel chip oil displacement experiments (large channel, 25 °C, deionized water): (a) Original oil-filled image; (b) After injection of acrylamide copolymer nanosphere emulsion dispersion; (c) After injection of copper quantum dot dispersion; Figure S4: Real-time images and blue channel histograms of microchannel chip oil displacement experiments(small channel, 25 °C, deionized water): (a) Original oil-filled image; (b) After injection of acrylamide copolymer nanosphere emulsion dispersion; (c) After injection of copper quantum dot dispersion; Figure S5: Real-time images and blue channel histograms of microchannel chip oil displacement experiments(small channel, 65°C, deionized water): (a) Original oil-filled image; (b) After injection of acrylamide copolymer nanosphere emulsion dispersion; (c) After injection of copper quantum dot dispersion; Figure S6: Real-time images and blue channel histograms of microchannel chip oil displacement experiments (large channel, 25 °C, injected water): (a) Original oil-filled image; (b) After injection of acrylamide copolymer nanosphere emulsion dispersion; (c) After injection of copper quantum dot dispersion.
