# Supplementary material for: Copper Quantum Dot/Polyacrylamide Composite Nanospheres: Spreading on Quartz Flake Surfaces and Displacing Crude Oil in Microchannel Chips
Source: Polymers (Basel). 2024 Apr 12;16(8):1085. doi: 10.3390/polym16081085 (PMC11053435; doi:10.3390/polym16081085)
Supplement: Supplementary file 1 [file polymers-16-01085-s001.zip › polymers-2942661-supplementary.pdf]

Supplementary Material for

Copper quantum dot/polyacrylamide composite nanospheres: spreading on quartz flake surfaces and displacing crude oil in microchannel chips

Xinru Ma [1,†], Haien Yang [2,†], Xiaofei Liu [1,*], Lixiang Zeng [2], Xinzi Li [1], Lijun Zheng [2], Yu Yang [1], Lei Cao [1], Weikai Meng [1] and Junping Zheng [1]

[1] Tianjin Key Laboratory of Composite and Functional Materials, School of Material Science and Engineering, Tianjin University, Tianjin, 300072, China

[2] Xi'an Changqing Chemical Industry Group Co., Ltd, Xi'an, 710021, China

* Email: liuxf315@aliyun.com

† These authors contributed equally to this work.

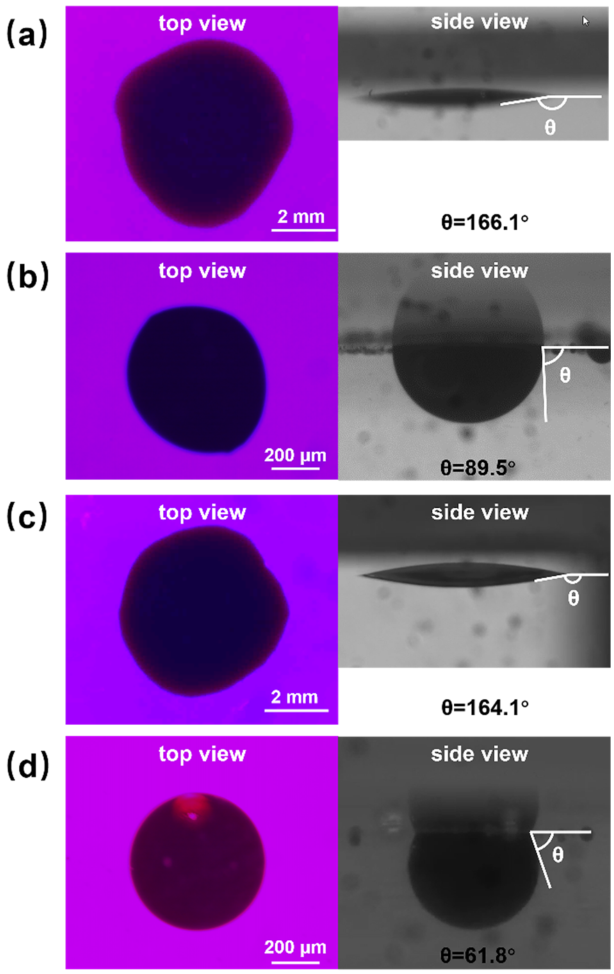

Figure S1. Images of 0.1 wt% acrylamide copolymer nanospheres dry powder dispersion at different temperatures for nanofluid spreading experiments: (**a**) 25°C, experimental initial plots; (**b**) 25°C, experimental 60 min effect plots; (**c**) 65°C, experimental initial plots; (**d**) 65°C, experimental 60 min effect plots.

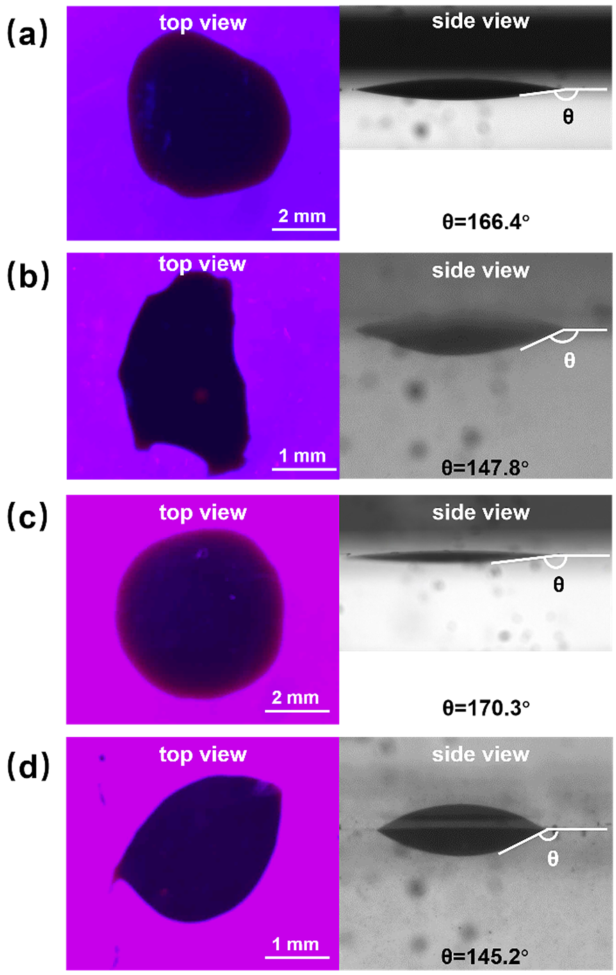

Figure S2. Images of 100 ppm copper quantum dot dispersion at different temperatures for nanofluid spreading experiments: (**a**) 25°C, experimental initial plots; (**b**) 25°C, experimental 60 min effect plots; (**c**) 65°C, experimental initial plots; (**d**) 65°C, experimental 60 min effect plots.

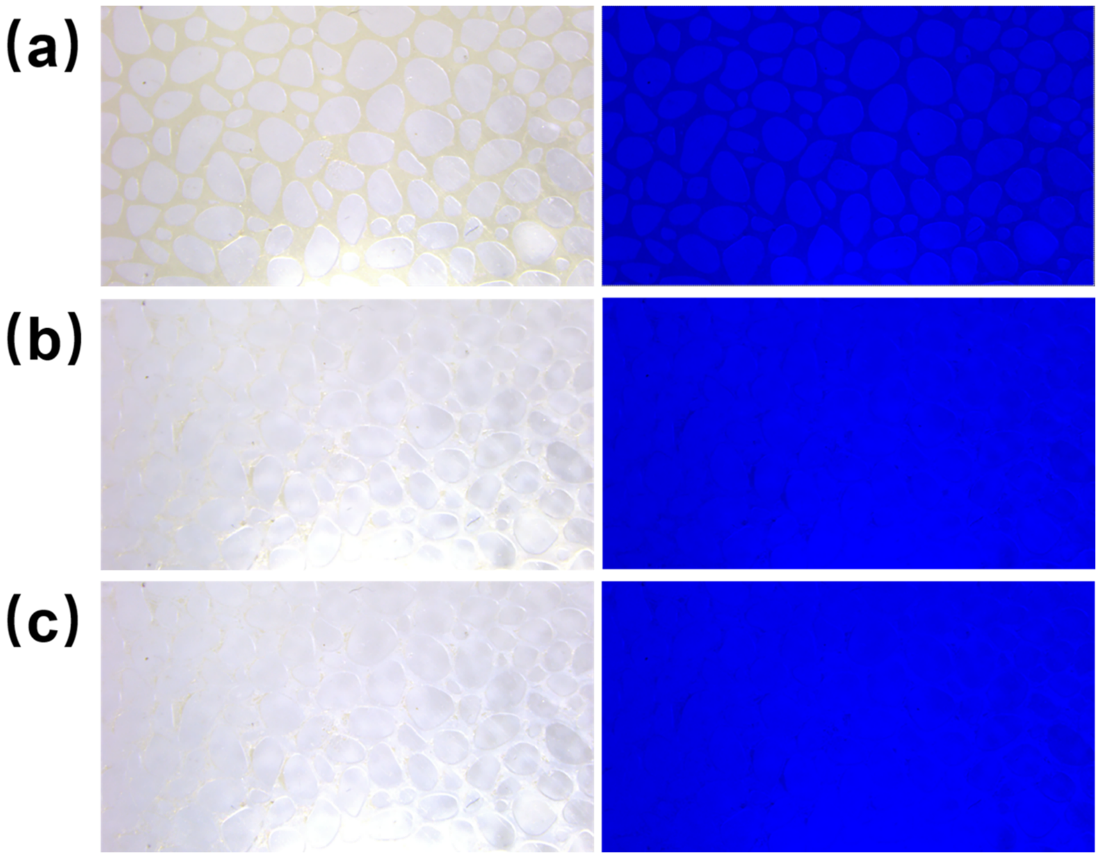

Figure S3. Real-time images and blue channel histograms of microchannel chip oil displacement experiments (large channel, 25°C, deionized water): (**a**) Original oil-filled image; (**b**) After injection of acrylamide copolymer nanosphere emulsion dispersion; (**c**) After injection of copper quantum dot dispersion.

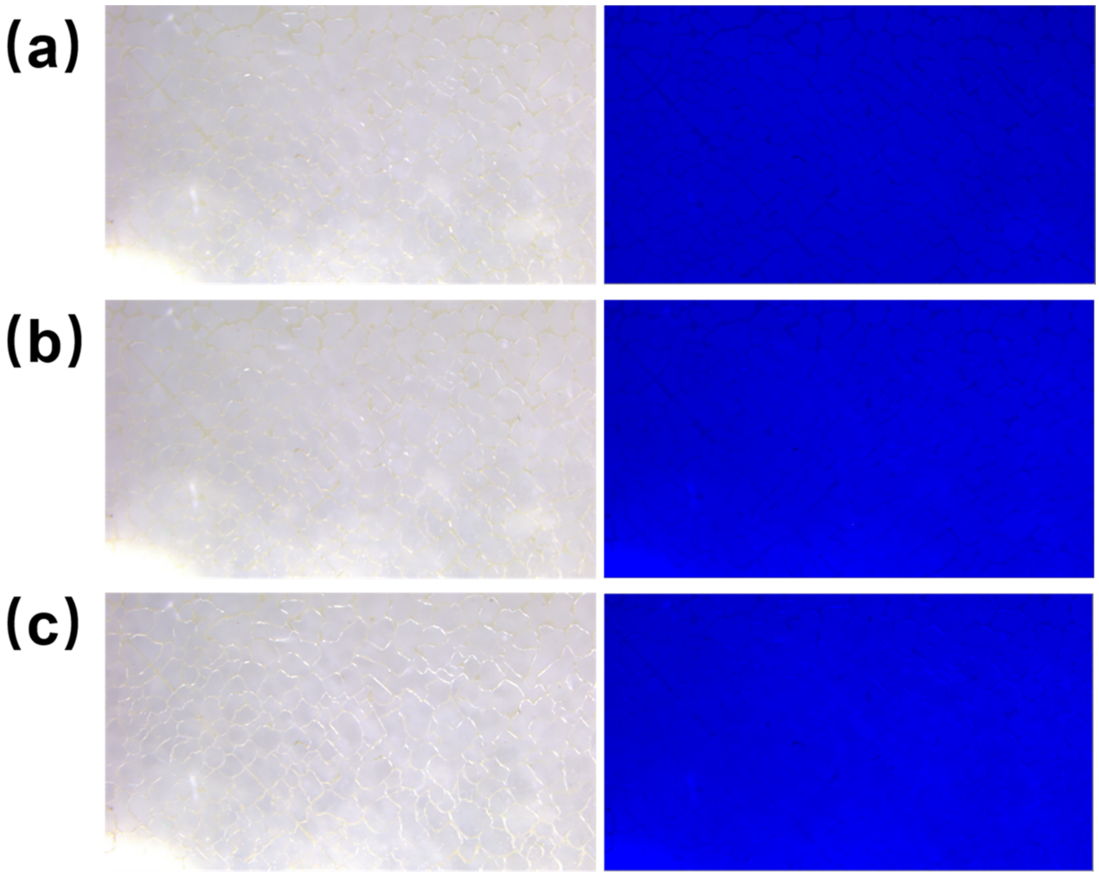

Figure S4. Real-time images and blue channel histograms of microchannel chip oil displacement experiments(small channel, 25°C, deionized water): (**a**) Original oil-filled image; (**b**) After injection of acrylamide copolymer nanosphere emulsion dispersion; (**c**) After injection of copper quantum dot dispersion.

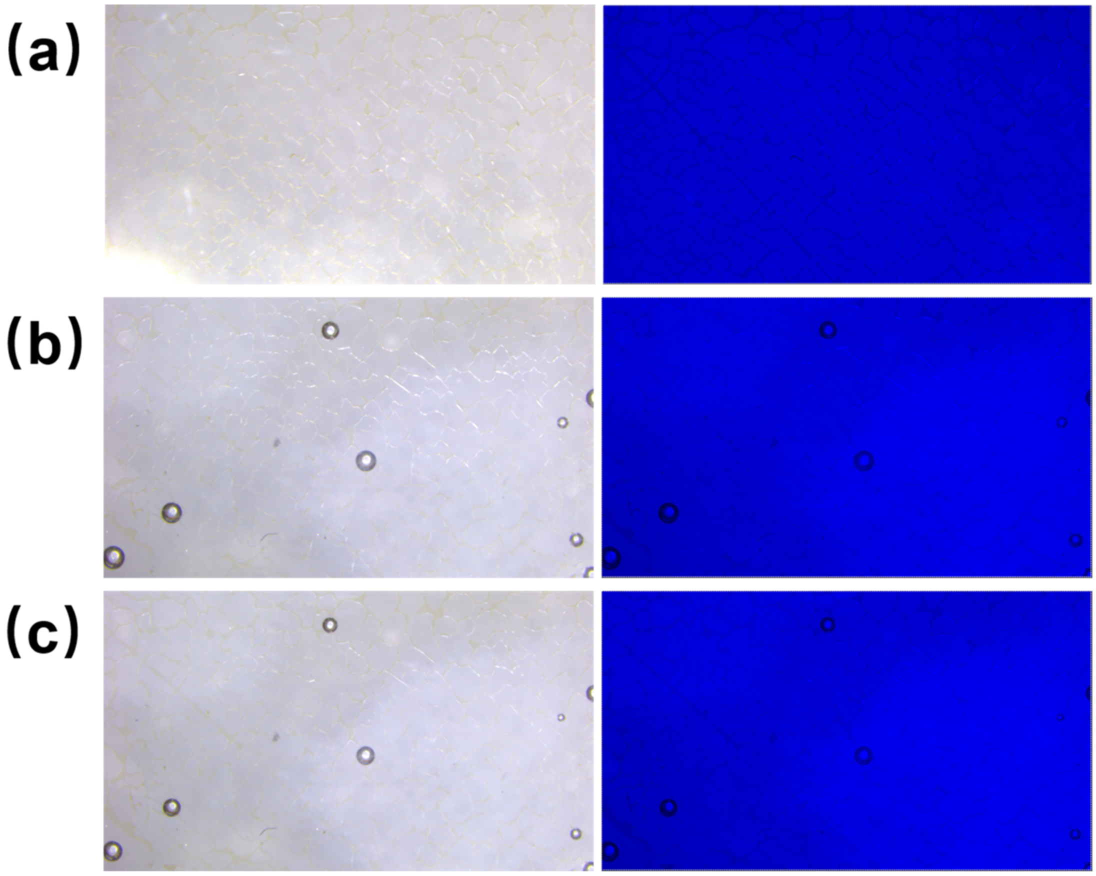

Figure S5. Real-time images and blue channel histograms of microchannel chip oil displacement experiments(small channel, 65°C, deionized water): (**a**) Original oil-filled image; (**b**) After injection of acrylamide copolymer nanosphere emulsion dispersion; (**c**) After injection of copper quantum dot dispersion.

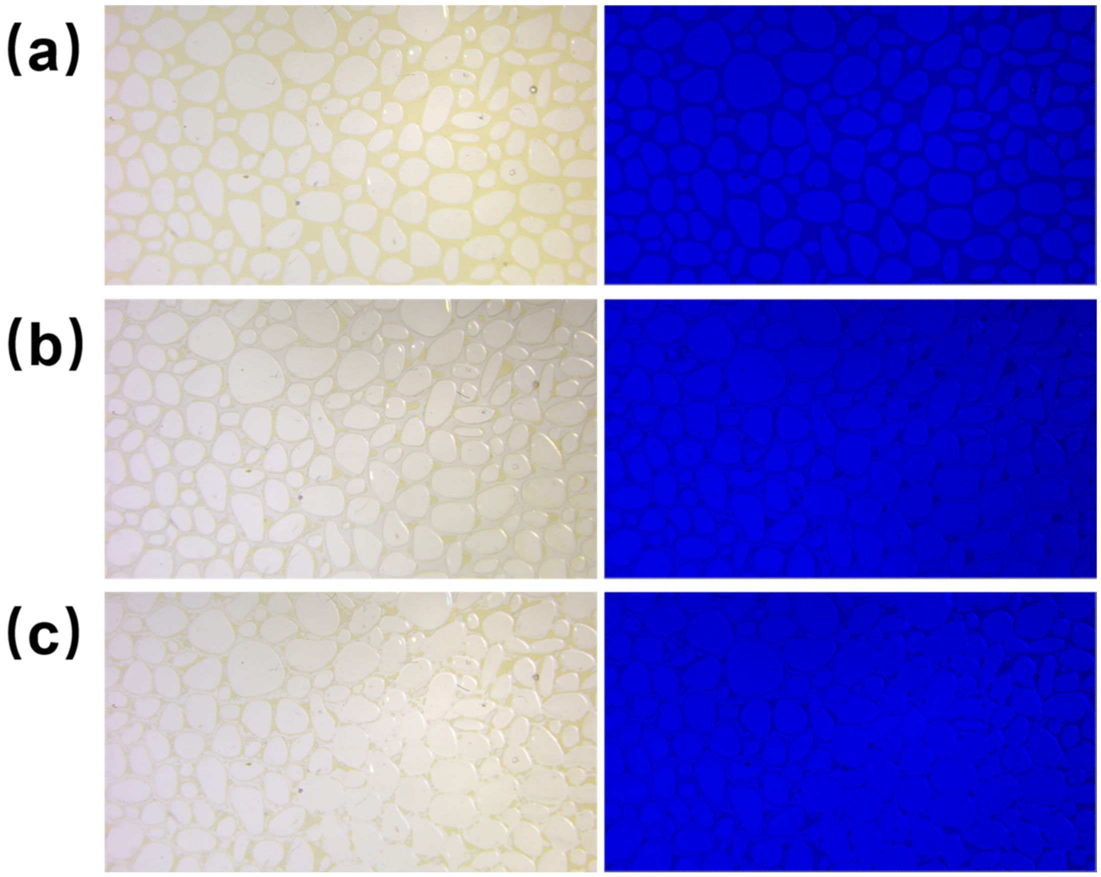

Figure S6. Real-time images and blue channel histograms of microchannel chip oil displacement experiments (large channel, 25°C, injected water): (**a**) Original oil-filled image; (**b**) After injection of acrylamide copolymer nanosphere emulsion dispersion; (**c**) After injection of copper quantum dot dispersion.
